# Supplementary material for: Non-collinear interaction of photons with orbital angular momentum
Source: Sci Rep. 2013 Dec 13;3:3491. doi: 10.1038/srep03491 (PMC3861806; doi:10.1038/srep03491)
Supplement: Supplementary Information — Non-collinear interaction of photons with orbital angular momentum [file srep03491-s1.pdf]

Supplementary Information: Non-collinear interaction of photons with orbital angular momentum

T. Roger, J. F. Heitz, D. Faccio and E. M. Wright

November 12, 2013

This supplementary information provides the full derivation of non-collinear phase matching equations shown in the main text.

1 Basic geometry and equations

1.1 Propagation equations

We consider type I phase-matching in a uniaxial crystal. The two fundamental fields of frequency ω and traveling at small angles $\pm\theta$ with respect to the z-axis are labeled $j = 0, 1$ are o-waves, and the second-harmonic is an e-wave labeled $j = 2$ which propagates along the z-axis. We employ the undepleted pump beam and paraxial approximations providing the equations for the fundamental fields as [1]

$$\frac{\partial E_j}{\partial z} = \frac{i}{2k_o}\nabla_\perp^2 E_j, \quad j = 0, 1 \tag{1}$$

with $k_o = \omega n_o/c$ being the magnitude of the o-wave wave vector, and $\nabla_\perp^2$ is the transverse Laplacian describing beam diffraction. In a similar manner the equation for the second-harmonic is

$$\frac{\partial E_2}{\partial z} = \frac{i}{2k_e}\nabla_\perp^2 E_2 + i\eta E_0(\vec{r})E_1(\vec{r})e^{i\Delta k(0)z}, \tag{2}$$

where $k_e = 2\omega n_e/c$ is the magnitude of the e-wave wave vector, $\eta = 32\pi d_{eff}\omega^2/k_e c^2$ with d_{eff} the nonlinear coefficient, and $\Delta k(0) = (2k_o - k_e)$ is the plane-wave wave vector mismatch projected along the z-axis. There is no incident SHG so $E_2(x, y, 0) = 0$.

1.2 Fundamental beam properties

We specifically consider the case of BBO and two fundamental fields propagating at small angles $\pm\theta$ with respect to the z-axis. Each fundamental field may be either a Gaussian or a field with donut shaped intensity profile that carries OAM with winding number ℓ. The transverse spatial extent of both fundamental beams is assumed so large that diffraction may be safely neglected within the medium, that is, their Rayleigh ranges

are much larger than the medium length L. For the OAM beam propagating along the z-axis the local wave vector around the ring may be written as

$$\begin{aligned}\vec{K} &= K_x\vec{e}_x + K_y\vec{e}_y + K_z\vec{e}_z \\ &= \frac{\ell}{R}\cos(\phi)\vec{e}_x + \frac{\ell}{R}\sin(\phi)\vec{e}_y + K_z\vec{e}_z,\end{aligned} \tag{3}$$

with $R >> \lambda$ the ring radius, and ϕ the azimuthal angle around the ring. By demanding that $K^2 = k_0^2$ we obtain for a forward propagating field

$$\begin{aligned}K_z &= \sqrt{k_o^2 - \frac{\ell^2}{R^2}} \\ &\approx k_o - \frac{1}{2k_o}\frac{\ell^2}{R^2},\end{aligned} \tag{4}$$

so we get the expected reduction in the z-component of the wave vector due to the skewing associated with the OAM.

If we rotate this wave vector by a small angle $|\theta| << 1$ around an axis that here we take as the y-axis, then the components of the wave vector become to leading order

$$\begin{aligned}K'_x &= K_x\cos(\theta) - K_z\sin(\theta) \approx \frac{\ell}{R}\cos(\phi) - k_o\theta, \\ K'_y &= K_y \approx \frac{\ell}{R}\sin(\phi), \\ K'_z &= K_z\cos(\theta) + K_x\sin(\theta) \approx k_o - \frac{1}{2k_o}\frac{\ell^2}{R^2} + \frac{\ell\theta}{R}\cos(\phi).\end{aligned} \tag{5}$$

For the rotated wave vector $\vec{K}'$ the associated plane-wave is

$$e^{i\vec{K}'\cdot\vec{r}} \to e^{iK'_z z}e^{-ik_0\theta x}e^{i\ell\phi}. \tag{6}$$

So as a result of the rotation we have a modification of the z-component of the wave vector, a tilt factor along the x-axis, along with the original OAM factor $\exp(i\ell\phi)$.

Based on the above discussion we write the fundamental field labeled $j = 0$, which makes a small angle θ with respect to the z-axis, in the form

$$E_0(\vec{r}) = \begin{cases} A_G(\rho)e^{ik_0\theta x} \\ A_\ell(\rho)e^{i(K'_z-k_o)z}e^{ik_0\theta x}e^{i\ell\phi}\end{cases} \tag{7}$$

depending on whether the field is a Gaussian or OAM beam. Here we have neglected any z-variation of the fundamental field envelopes A_G and A_ℓ based on the fact that the Rayleigh ranges of the fundamental fields are much larger than the medium length. Similarly we write the fundamental field labeled $j = 1$, which makes a small angle $-\theta$ with respect to the z-axis, in the form

$$E_1(\vec{r}) = \begin{cases} A_G(\rho)e^{-ik_0\theta x} \\ A_\ell(\rho)e^{i(K'_z-k_o)z}e^{-ik_0\theta x}e^{i\ell\phi}\end{cases} \tag{8}$$

where $A_G(\rho)$ and $A_\ell(\rho) = A_{-\ell}(\rho)$ are the radial distributions of the Gaussian and OAM fundamental beams, ρ being the radial coordinate in cylindrical components.

1.3 Nonlinear polarization

To proceed let us inspect the nonlinear polarization source term for SHG involving $E_0(\vec{r})E_1(\vec{r})$ on the right-hand-side of Eq. (2). We shall consider three specific cases that cover the various options of fundamental fields. Case (i) involves one Gaussian beam and one OAM beam with winding number ℓ

$$E_0(\vec{r}) = A_G(\rho)e^{ik_0\theta x}, \quad E_1(\vec{r}) = A_\ell(\rho)e^{i(K_z'-k_o)z}e^{-ik_0\theta x}e^{i\ell\phi}. \tag{9}$$

We then find

$$\begin{aligned} E_0(\vec{r})E_1(\vec{r}) &= A_G(\rho)A_\ell(\rho)\exp\left[i\ell\phi - \frac{iz}{2k_o}\frac{\ell^2}{R^2} + \frac{iz\ell\theta}{R}\cos(\phi)\right] \\ &= A_G(\rho)A_\ell(\rho)\sum_{n=-\infty}^{\infty} i^n \exp\left[i(\ell+n)\phi - \frac{iz}{2k_o}\frac{\ell^2}{R^2}\right]\mathrm{J_n}\left(\frac{z\ell\theta}{R}\right), \end{aligned} \tag{10}$$

where we have used the Jacobi-Anger expansion in the last line. The nonlinear polarization will therefore contain a range of winding numbers for non-zero angle θ. For the moment we concentrate on the case $n = 0$ corresponding to SHG with the same winding number ℓ as the input fundamental. (This is justified if the argument $|z\ell\theta/R| << 1$ remains small since $\mathrm{J_n}(s) \to 0$ for $n \neq 0$ and small $|s|$.) We then have

$$E_0(\vec{r})E_1(\vec{r}) \to A_G(\rho)A_\ell(\rho)e^{\left[i\ell\phi - \frac{iz}{2k_o}\frac{\ell^2}{R^2}\right]}\mathrm{J_0}\left(\frac{z\ell\theta}{R}\right), \tag{11}$$

and for case (i) the paraxial wave Eq. (2) for the SHG field becomes

$$\frac{\partial E_2}{\partial z} = \frac{i}{2k_e}\nabla_\perp^2 E_2 + i\eta A_G(\rho)A_\ell(\rho)e^{\left[i\ell\phi + i\left(\Delta k(0) - \frac{1}{2k_o}\frac{\ell^2}{R^2}\right)z\right]}\mathrm{J_0}\left(\frac{z\ell\theta}{R}\right), \tag{12}$$

and the generated noncollinear SHG will have winding number ℓ. We note that the right-hand-side contains both an exponential term, which is familiar from conventional phase-matching, along with a Bessel term arising from the term linear in ℓ in the wave vector in Eq. (5) of the tilted OAM beam.

One can easily repeat this procedure for the other cases. For example, case (ii) involves two fundamental OAM fields with opposite winding number $\pm\ell$ so that

$$\begin{aligned} E_0(\vec{r})E_1(\vec{r}) &= A_\ell^2(\rho)\exp\left[-\frac{iz}{k_o}\frac{\ell^2}{R^2} + \frac{2iz\ell\theta}{R}\cos(\phi)\right] \\ &= A_\ell^2(\rho)\sum_{n=-\infty}^{\infty} i^n \exp\left[in\phi - \frac{iz}{k_o}\frac{\ell^2}{R^2}\right]\mathrm{J_n}\left(\frac{2z\ell\theta}{R}\right), \end{aligned} \tag{13}$$

which upon restricting to the $n = 0$ term yields

$$E_0(\vec{r})E_1(\vec{r}) \to A_\ell^2(\rho)e^{\left[-\frac{iz}{k_o}\frac{\ell^2}{R^2}\right]}\mathrm{J_0}\left(\frac{2z\ell\theta}{R}\right). \tag{14}$$

For case (ii) the paraxial wave equation for the SHG field becomes

$$\frac{\partial E_2}{\partial z} = \frac{i}{2k_e}\nabla_\perp^2 E_2 + i\eta A_\ell^2(\rho)e^{\left[i\left(\Delta k(0)-\frac{1}{k_o}\frac{\ell^2}{R^2}\right)z\right]}\mathrm{J}_0\left(\frac{2z\ell\theta}{R}\right), \tag{15}$$

meaning that the generated non-collinear SHG will have zero winding number. Both the exponential and Bessel terms appear on the right-hand-side again.

In contrast, case (iii) involves two OAM fundamental fields with the same winding number ℓ for which we find

$$E_0(\vec{r})E_1(\vec{r}) \to A_\ell^2(\rho)e^{\left[2i\ell\phi-\frac{iz}{k_o}\frac{\ell^2}{R^2}\right]}, \tag{16}$$

so that the paraxial wave equation for the SHG field for case (iii) becomes

$$\frac{\partial E_2}{\partial z} = \frac{i}{2k_e}\nabla_\perp^2 E_2 + i\eta A_\ell^2(\rho)e^{\left[2i\ell\phi+i\left(\Delta k(0)-\frac{1}{k_o}\frac{\ell^2}{R^2}\right)z\right]}, \tag{17}$$

meaning that the generated noncollinear SHG will have winding number 2ℓ. We note that the Bessel term does not appear in case (iii).

2 Noncollinear second-harmonic generation

Here we examine the three cases of noncollinear SHG alluded to above.

2.0.1 Case (i)

Based on Eq. (12) we write the second harmonic field as $E_2(\vec{r}) = \mathcal{E}_2(\rho, z)e^{i\ell\phi}$ which yields

$$\frac{\partial \mathcal{E}_2}{\partial z} = \frac{i}{2k_e}\left(\frac{\partial^2}{\partial\rho^2}+\frac{1}{\rho}\frac{\partial}{\partial\rho}-\frac{\ell^2}{\rho^2}\right)\mathcal{E}_2 + i\eta A_G(\rho)A_\ell(\rho)e^{\left[i\left(\Delta k(0)-\frac{1}{2k_o}\frac{\ell^2}{R^2}\right)z\right]}\mathrm{J}_0\left(\frac{z\ell\theta}{R}\right). \tag{18}$$

In the geometry considered the OAM beam is a thin ring of radius $R >> \lambda$ and thickness $R > W >> \lambda$, and the medium is much thinner than the Rayleigh range of the propagating fields. We thus drop the radial derivatives describing beam diffraction but retain the term varying as ℓ^2/ρ^2.

Hereafter we evaluate the SHG at the peak of the ring $\rho = R$, and the SHG power is evaluated as $P_2 = 2\pi R \cdot W|E_2(R,z)|^2$. To proceed we set $\mathcal{E}_2(\rho = R, z) = A_2(z)e^{-\frac{iz}{2k_e}\frac{\ell^2}{R^2}}$ leading to

$$\frac{dA_2}{dz} = i\eta A_G(R)A_\ell(R)e^{i\Delta k(\ell)z}\mathrm{J}_0\left(\frac{z\ell\theta}{R}\right), \tag{19}$$

with $A_\ell(R)$ the field value of the OAM field around the ring which is taken independent of ℓ, and wave vector mismatch

$$\Delta k(\ell) = \Delta k(0) + \frac{1}{2}\left(\frac{1}{k_e}-\frac{1}{k_o}\right)\frac{\ell^2}{R^2}. \tag{20}$$

We assume that the SHG is phase-matched for $\ell = 0$ which requires $\Delta k(0) = (2k_0 - k_e) = 0$, we have

$$\Delta k(\ell) = -\frac{1}{4k_o}\frac{\ell^2}{R^2}, \tag{21}$$

giving the final equation for the SHG

$$\frac{dA_2}{dz} = C \cdot e^{-\frac{iz}{4k_o}\frac{\ell^2}{R^2}} \mathrm{J}_0\left(\frac{z\ell\theta}{R}\right), \tag{22}$$

with $C = i\eta A_0(R)A_\ell(R)$.

2.0.2 Phase-matching

We shall consider the effects of the exponential and Bessel terms separately to assess their roles. Consider first the limit $\theta \to 0$ so that only the exponential term is present. Then for a fixed crystal length L the SHG power varies as

$$P_2(\ell) \propto \mathrm{sinc}^2\left(\frac{\ell^2}{16\pi}\frac{\lambda_1 L}{n_o R^2}\right), \tag{23}$$

with λ_1 the fundamental wavelength. The phase-matching for $\ell = 0$ is thus destroyed for $|\ell| > \ell_C$ with $\ell_C = 4\pi R\sqrt{n_o/\lambda_1 L}$. Consider next the Bessel term alone then

$$A_2(L) = C\int_0^L dz \mathrm{J}_0\left(\frac{z\ell\theta}{R}\right). \tag{24}$$

A closed form solution for this integral may be expressed in terms of Bessel and Struve functions, but as long as the argument of the Bessel function in the integrand remains small compared to unity we obtain

$$P_2(\ell) \propto \left(1 - \frac{\ell^2}{\ell_c^2}\right), \tag{25}$$

with $\ell_c \approx |\sqrt{6}R/\theta L|$. By comparing ℓ_C and ℓ_c we can see which dominates in a given situation. We also note that an expression for the SHG power of the same form as above arises in the treatment of walk-off effects and beam tilt due to the SLM but with θ in the expression for ℓ_c replaced by the walk-off angle or tilt angle.

We note that the ratio of the two critical winding numbers is independent of R

$$r = \frac{\ell_C}{\ell_c} \simeq 5|\theta|\sqrt{\frac{Ln_o}{\lambda_1}}, \tag{26}$$

and the Bessel term will dominate over the exponential term if $r >> 1$ and vice versa. We note that for small enough angles $|\theta| \to 0$ then $r \to 0$ so the exponential term will always dominate. For the parameters of the experiment we find $r \geq 5$ so the Bessel term should indeed dominate.

2.0.3 Case (ii)

Based on Eq. (15) we write the second harmonic field as $E_2(\vec{r}) = \mathcal{E}_2(\rho, z)$ which yields

$$\frac{\partial \mathcal{E}_2}{\partial z} = \frac{i}{2k_e}\left(\frac{\partial^2}{\partial \rho^2} + \frac{1}{\rho}\frac{\partial}{\partial \rho}\right)\mathcal{E}_2 + i\eta A_\ell^2(\rho) e^{\left[i\left(\Delta k(0) - \frac{1}{k_o}\frac{\ell^2}{R^2}\right)z\right]} \mathrm{J}_0\left(\frac{2z\ell\theta}{R}\right). \quad (27)$$

As before for the chosen geometry we drop the radial derivatives describing beam diffraction. To proceed we set $\mathcal{E}_2(\rho = R, z) = A_2(z)$ at the peak of the ring leading to

$$\frac{dA_2}{dz} = Ce^{i\Delta k(\ell)z}\mathrm{J}_0\left(\frac{2z\ell\theta}{R}\right), \quad (28)$$

with $C = i\eta A_\ell^2(R)2$, and wave vector mismatch

$$\Delta k(\ell) = \Delta k(0) - \frac{1}{k_o}\frac{\ell^2}{R^2}. \quad (29)$$

2.0.4 Phase-matching

In this case two fundamentals of opposite OAM combine to make an SHG field of zero winding number. This case is therefore the reverse process of parametric down conversion so lessons learned here apply to PDC also [2, 3]. In particular, notice that the propagation equation involves not only the conventional phase-matching described by the exponential term but also has the Bessel term. To the best of our knowledge the Bessel term has not appeared in previous treatments of PDC, perhaps justified but the fact that small angles are employed.

In the limit $\theta \to 0$ only the ℓ^2 term remains and this is the usual assumption in PDC. In that limit if the SHG is phase-matched for $\ell = 0$ which requires $\Delta k(0) = (2k_0 - k_e) = 0$, the SHG power varies as

$$P_2(\ell) \propto \mathrm{sinc}^2\left(\frac{\ell^2}{4\pi}\frac{\lambda_1 L}{n_o R^2}\right). \quad (30)$$

The phase-matching for $\ell = 0$ is thus destroyed for $|\ell| > \ell_C$ with $\ell_C = 2\pi R\sqrt{n_o/\lambda_1 L}$. Consider next the Bessel term alone, then the SH field varies as

$$A_2(L) = C\int_0^L dz \mathrm{J}_0\left(\frac{2z\ell\theta}{R}\right). \quad (31)$$

This may be simplified if the Bessel function is not too large compared to unity, then we obtain

$$P_2(\ell) \propto \left(1 - \frac{\ell^2}{\ell_c^2}\right), \quad (32)$$

with $\ell_c \approx |\sqrt{6}R/2\theta L|$.

We note that the ratio of the two critical winding numbers is independent of R

$$r = \frac{\ell_C}{\ell_c} \simeq 5|\theta|\sqrt{\frac{Ln_o}{\lambda_1}}, \quad (33)$$

and the Bessel term will dominate over the regular phase-matching term if $r >> 1$ and vice versa.

2.0.5 Case (iii)

Based on Eq. (17) we write the second harmonic field as $E_2(\vec{r}) = \mathcal{E}_2(\rho, z)e^{2i\ell\phi}$ which yields

$$\frac{\partial \mathcal{E}_2}{\partial z} = \frac{i}{2k_e}\left(\frac{\partial^2}{\partial \rho^2} + \frac{1}{\rho}\frac{\partial}{\partial \rho} - \frac{4\ell^2}{\rho^2}\right)\mathcal{E}_2 + i\eta A_\ell^2(\rho)e^{\left[i\left(\Delta k(0) - \frac{1}{k_o}\frac{\ell^2}{R^2}\right)z\right]}. \tag{34}$$

For the geometry considered we drop the radial derivatives describing beam diffraction but retain the term including $4\ell^2/R^2$.

To proceed we set $\mathcal{E}_2(\rho = R, z) = A_2(z)e^{-\frac{2iz}{k_e}\frac{\ell^2}{R^2}}$ at the ring peak leading to

$$\frac{dA_2}{dz} = Ce^{i\Delta k(\ell)z}, \tag{35}$$

with $C = iA_\ell^2(R)$, and wave vector mismatch

$$\Delta k(\ell) = \Delta k(0) + \left(\frac{2}{k_e} - \frac{1}{k_o}\right)\frac{\ell^2}{R^2}. \tag{36}$$

2.0.6 Phase-matching

For a fixed medium length the SH power will vary with the winding number ℓ as

$$P_2(\ell) \propto \mathrm{sinc}^2\left(\frac{\Delta k(\ell)L}{2}\right). \tag{37}$$

if we assume that the SHG is phase-matched for $\ell = 0$ which requires $\Delta k(0) = (2k_0 - k_e) = 0$, then using $k_e = 2k_o$ we find $\Delta k(\ell) = 0$. This is in keeping with the known result for single beam SHG that if the integration is phase-matched for a Gaussian beam it is also phase-matched for OAM beams of varying winding number [4].

References

[1] See, for example, R. W. Boyd, *Nonlinear Optics*.

[2] J. P. Torres, A. Alexandrescu, and L. Torner, *Phys. Rev. A* **68**, 050301(R) (2003).

[3] F. M. Miatto, A.M. Yao, and S. M. Barnett, *Phys. Rev. A* **83**, 033816 (2011).

[4] K. Dholakia, N. B. Simpson, M. J. Padgett, and L. Allen, *Phys. Rev. A* **54**, R3742 (1996).
